# Hypomagnetic Field Enhances U2OS Cell Proliferation and Migration by Promoting β-Catenin Phosphorylation and Upregulating FN1 and LOX Expression

**DOI:** 10.3390/cells15080727

**Published:** 2026-04-19

**Authors:** Taotao Gao, Wenfeng Zhong, Mengli Tao, Yu Guo, Kun Yang, Yaohui He, Guosheng Hu, Long Li, Xiangyan Kong, Fulai Li, Yufen Zhao

**Affiliations:** 1 Institute of Drug Discovery Technology, Ningbo University, Ningbo 315211, China; 13003701935@163.com (T.G.); 2311390163@nbu.edu.cn (M.T.); 19834219609@163.com (Y.G.); zhaoyufen@nbu.edu.cn (Y.Z.); 2 Qian Xuesen Collaborative Research Center of Astrochemistry and Space Life Sciences, Ningbo University, Ningbo 315211, China; 3 Biomedical Research Center of South China, College of Life Sciences, Fujian Normal University, Fuzhou 350117, China; wenfengzhong0604@163.com (W.Z.); hugs314@163.com (G.H.); 4 Faculty of Electrical Engineering and Computer Science, Institute for Future Wireless Research (IFWR), Ningbo University, Ningbo 315211, China; 2201100027@nbu.edu.cn (K.Y.); kongxiangyan@nbu.edu.cn (X.K.); 5 MOE Key Lab of Rare Pediatric Diseases, Hengyang Medical School, University of South China, Hengyang 421001, China; 2024001050@usc.edu.cn

**Keywords:** hypomagnetic field (HMF), biological effects, protein phosphorylation, β-Catenin, LOX, FN1, proliferation and migration

## Abstract

Accumulating evidence indicates that a hypomagnetic field (HMF, <5 μT) has a significant impact on various organ systems in animals. However, the cellular and molecular mechanisms underlying these biological effects remain unclear. Understanding the molecular mechanisms underlying mammalian responses to a HMF is crucial for addressing health and safety concerns associated with HMF exposure. In this study, we investigated the changes in intracellular protein phosphorylation under HMF conditions and validated the functional mechanisms by which HMF-induced protein phosphorylation affects cell behavior. We found that U2OS cells can rapidly sense changes in magnetic fields, leading to alterations in protein phosphorylation levels within the cell. The quantitative phosphoproteomics results revealed that the exposure of U2OS cells to the HMF environment for 0.5 h and 3 days resulted in the alteration of 1101 and 1543 phosphosites, respectively. Notably, HMF exposure enhanced the phosphorylation of β-Catenin at Ser552, and this increased phosphorylation-promoted U2OS proliferation and migration. Furthermore, quantitative proteomics showed that exposure to a HMF for 3 days upregulated the expression of LOX and FN1, while the knockdown of LOX or FN1 suppressed the proliferation and migration of the U2OS cells. These results suggest that a HMF enhances U2OS cell proliferation and migration by promoting β-Catenin phosphorylation and upregulating FN1 and LOX expression.

## 1. Introduction

Manned deep space exploration and the search for habitable exoplanets have long been a significant aspiration of humanity. However, once astronauts venture into space, they inevitably expose themselves to a unique environment characterized by space radiation, microgravity conditions, a closed habitat, confinement, and considerable distance from Earth [1]. Moreover, the weakening and/or absence of the magnetic field has been identified as another environmental challenge that is often overlooked [2]. As a natural component of the environment, the geomagnetic field (GMF, ~50 μT) is crucial for the survival and evolution of life on Earth [3], meaning that humans have become accustomed to and dependent on the GMF [4]. However, an extremely weak magnetic field exists on the Moon and Mars, as well as in interstellar space [5]. During deep space flights and sojourns, astronauts will experience a hypomagnetic field (HMF, <5 μT), which poses a critical problem to astronauts during long-term interplanetary missions and may jeopardize their health. Consequently, research on the biological effects of HMFs, particularly concerning health and safety issues that astronauts may encounter during prolonged space flights, has garnered significant attention regarding the impacts induced by HMFs.

In recent decades, research on the biological effects of HMFs has significantly expanded, revealing its substantial impact on various organ systems in animals, including reproductive and embryonic development, the cardiovascular system, and the central nervous system, as well as related behaviors [6,7]. The effects of HMFs on animal embryos and their development processes are particularly noteworthy, with most of these impacts being negative in nature. Studies indicated that HMF exposure negatively influences development, leading to a notable increase in the frequency of developmental blockages and abnormalities in Japanese newts and *Xenopus* [8,9]. Fesenko et al. [10] found that HMF exposure adversely affects reproductive function; pregnant mice exposed to HMF for 3 to 12 days lost their ability to bear offspring. Additional research suggests that HMF exposure may disrupt the physiological rhythms of animals and related behaviors in animals [11,12]. Mo et al. [13] observed that HMF-exposed animals exhibited a prolonged alteration of the circadian drinking rhythm and a decrease in general activity, accompanied by an increase in thermal hyperalgesia. Furthermore, a HMF is also reported to have detrimental effects on the skeletal and immune systems [14,15,16]. Recent studies have demonstrated that a HMF significantly impacts the nervous system of animals, influencing cognitive processes in humans as well as adult hippocampal neurogenesis and cognitive behaviors in mice [17,18,19]. Despite the growing documentation of the biological effects of HMF, the cellular and molecular mechanisms underlying these effects remain unclear.

Understanding the molecular mechanisms underlying mammalian responses to HMFs is not only a critical issue in magnetobiology but also provides an essential experimental basis for safeguarding the health and safety of astronauts during space exploration. Protein phosphorylation is a well-established post-translational modification that occurs ubiquitously in biological systems and is integral to nearly all cellular life processes. This modification regulates essential biological functions, including growth, development, aging, apoptosis, signal transduction, as well as the onset and progression of various diseases [20]. Protein phosphorylation is dynamic, reversible, and is regulated by specific protein kinases and phosphatases. In mammalian cells, over 500 protein kinases and approximately 200 phosphatases that facilitate the dynamic phosphorylation process have been identified [21,22]. The dynamic and reversible nature of protein phosphorylation can induce changes in protein structure, thereby influencing the protein’s biological activity, intracellular localization, and interactions with other molecules or cellular structures. Protein phosphorylation modification serves as a vital regulatory mechanism through which cells respond to environmental stress or changes in their external surroundings, such as temperature, microgravity and radiation [23,24,25]. The GMF serves as a crucial external factor for the survival of organisms and cells, and the alteration in its strength essentially constitutes an external environmental stimulus. Compared to the GMF, the intensity of the magnetic field in a HMF environment is significantly reduced (changes of three orders of magnitude). Therefore, protein phosphorylation modifications are likely to play a significant role in regulating cellular responses to a HMF environment.

In the present study, we found that the HMF in our research enhances the proliferation and migration of U2OS cells. We investigated the changes in intracellular protein phosphorylation modifications under HMF conditions and validated the functional mechanisms by which HMF-induced protein phosphorylation affects cell behavior. Our findings revealed that exposure of U2OS cells to the HMF environment for 0.5 h and 3 days resulted in significant changes in intracellular protein phosphorylation modifications. Notably, HMF exposure enhanced the phosphorylation of β-catenin at S552, and this increased the phosphorylation-promoted U2OS proliferation and migration. Furthermore, quantitative proteomics showed that exposure to the HMF for 3 days upregulated the expression of LOX and FN1. The knockdown of LOX or FN1 suppressed the proliferation and migration of the U2OS cells in both the GMF and the HMF. Collectively, these results indicate that the HMF environment facilitates the phosphorylation of β-catenin at Ser552 and the upregulation of expression of LOX and FN1, thereby promoting the proliferation and migration of U2OS cells.

## 2. Materials and Methods

### 2.1. Setups of Magnetic Field Conditions and Exposure Experiments

The HMF was achieved using a permalloy magnetic shield bucket, which included a central acrylic plate for dish placement, while maintaining HMF conditions (<50 nT) (Figure S1). The shielding bucket was placed inside a cell culture incubator (Heal Force). The cells for the GMF control were cultured in the same cell culture incubator, where the magnetic field was measured as approximately ~50 μT. The magnetic field was monitored using an axial fluxgate magnetometer.

### 2.2. MTS Assay, Clonal Formation

Cell proliferation was detected using the CellTiter 96^®^ AQueous one solution cell proliferation assay (Promega, Madison, WI, USA) following the manufacturer’s protocol. In brief, each well of the 96-well plates was seeded with cells at a density of 3000 cells per well. The plates were then placed in the cell culture incubator under the conditions of the GMF and the HMF, respectively. After 24, 48, 72 and 96 h, 10 μL of MTS solution was added to each well and incubated at 37 °C for 1 h. Subsequently, absorbance was measured at 490 nm using a SpectraMax Paradigm microplate reader (Molecular Devices, Shanghai, China). Each experiment was performed in triplicate.

For the colony formation assays, approximately 300 cells were seeded in a 6-well plate, and colonies were examined 14 days later. Briefly, colonies were fixed with methanol for 10 min and stained with 0.1% crystal violet for 15 min.

### 2.3. Cell Migration

The cells were seeded in a six-well plate. When the cells reached approximately 95% confluency, the medium was replaced with serum-free medium. Wounds were created in each well by scratching the monolayer culture using a sterile 200 μL micropipette tip. Then, the cells were carefully rinsed three times with PBS to remove cell debris. A fresh medium was put in place daily. The cell migration was monitored by capturing images immediately after wound creation and at given time points during wound closure. The wound area was analyzed, and the wound healing rate was quantified by comparing the wound area at specified time points to the initial wound area.

### 2.4. Protein Extraction, Reductive Alkylation and Trypsin Digestion

After HMF exposure, the cells were washed three times with pre-cold PBS. Subsequently, 1 mL of lysis buffer (100 mM Tris-HCl, 150 mM NaCl, 1% triton-X-100, 0.5% SDC, 0.1% SDS, 1 mM PMSF, 1 × complete EDTA-free protease inhibitor mixture (Roche, Mannheim, Germany), and 1 × phosSTOP phosphatase inhibitor mixture (Roche, Mannheim, Germany)) was immediately added to a 10 cm cell culture dish. The lysates from 4 cell culture dishes were collected in a 15 mL centrifuge tube and placed on ice for 10 min. Following lysis, the lysates were homogenized using a sonication probe for 5 min (45% of 150 W, with 5 s on and 25 s off) to solubilize the proteins and shear DNA. Finally, cell debris was removed by centrifugation at 12,000 *g* for 10 min at 4 °C. After debris removal, the supernatant was collected, and the total protein concentration was measured using the Bradford assay.

Next, 5 mg of proteins were transferred to an ultrafiltration centrifuge tube with a molecular weight cut-off of 10 kDa to further deplete the detrimental low-molecular-weight components and impurities. Following centrifugation, the supernatant was incubated with TCEP at room temperature at a final concentration of 30 mM for 10 min to reduce disulfide bonds. After reduction, the samples were centrifugated at 3800× *g* for 40 min, followed by alkylating with iodoacetamide at a final concentration of 50 mM. The samples were then centrifuged at 3800× *g* for 40 min and diluted with 50 mM ammonium bicarbonate (pH 8.0) until the urea concentration was reduced below 0.1 M. The total protein concentration was remeasured. Subsequently, the proteins were digested using trypsin (protein: trypsin = 100:1) for 3 h at 37 °C in an incubator. Followed by an overnight digestion again at the same ratios.

### 2.5. Desalting

Following digestion, the desalting process was performed. Briefly, samples were acidified using 10% formic acid (Sigma-Aldrich, Steinheim am Albuch, Germany) and centrifuged at 13,400× *g* for 10 min at 4 °C. The supernatant was then transferred to a tC18 Sep-Pak resin (Waters, Milford, MA, USA), which was activated and equilibrated with acetonitrile (ACN) and 0.1% formic acid, respectively. After loading the sample, the tC18 Sep-Pak resin was washed three times with 0.1% formic acid (2 mL), and the peptides were eluted with 50% ACN. The eluted peptides were then lyophilized to dryness and stored at −80 °C.

### 2.6. Phosphopeptides Enrichment

Phosphopeptides enrichment was performed using the High-Select™ Fe-NTA Phosphopeptide Enrichment Kit (Thermo Fisher Scientific™, Waltham, MA, USA) following the manufacturer’s recommended protocol. Briefly, lyophilized peptides were dissolved in 200 μL of binding/washing buffer and added to the equilibrated spin column. The samples were gently mixed by trapping the bottom plug for 10 s until the resin was in suspension. The samples were then incubated in the column for 30 min, with gentle mixing of the resin occurring every 10 min. Following incubation, unbound peptides were removed through centrifugation (1000× *g*, 30 s). Subsequently, the column was washed three times with 200 μL of binding/washing buffer. Prior to the elution step, the column was washed with 200 μL of LC-MS grade water via centrifugation (1000× *g*, 30 s). Finally, the enriched peptides were eluted twice with 100 μL elution buffer, and the eluted peptides were dried immediately in a speed vacuum concentrator.

### 2.7. LC-MS/MS Analysis

MS experiments were conducted using a nanoscale Vanquish Neo UHPLC system (Thermo Fisher Scientific, MA, USA) connected to an Orbitrap Exploris 480 MS equipped with a nanoelectrospray source (Thermo Fisher Scientific, MA, USA). Mobile phase A contained 0.1% formic acid (*v*/*v*) in water, while mobile phase B contained 0.1% formic acid in 80% ACN. The peptides were dissolved in 0.1% formic acid with 2% ACN and separated on an RP-HPLC analytical column (75 μm × 25 cm) packed with 2 μm C18 beads (Thermo Fisher Scientific). A linear gradient was applied, ranging from 8% to 28% ACN at 42 min, followed by a linear increase to 44% B at 9 min at a flow rate of 300 nL/min. The Orbitrap Exploris 480 MS acquired data in a data-independent manner, alternating between full scan MS and MS2 scans. The spray voltage was set to 2.2 kV, and the temperature of the ion transfer capillary was maintained at 300 °C. The MS spectra (380–980 m/z) were collected with a resolution of 120,000, an AGC target of 3 × 10^6^, and a maximal injection time of 25 ms. Ions were sequentially fragmented by HCD with 30% normalized collision energy, using specified isolation windows of 6.0 m/z, a resolution of 30,000, and a maximal injection time of 54 ms. Each sample was measured in triplicate.

### 2.8. Mass Spectrometry Data Analysis

The raw data were processed using DIA-NN (version 2.0), with MS/MS spectra searched against the SwissProt human database (downloaded in April 2024), which contains a total of 20,354 entries. All searches were carried out with a precursor mass tolerance of 10 ppm, oxidation (Met) (+15.9949 Da), carbamidomethylation (Cys) (+57.0215 Da), and acetylation (protein N-terminus) (+42.0106 Da) as modifications, allowing for two missed cleavages by trypsin. The peptide and protein identifications were filtered by DIA-NN to control the false discovery rate (FDR) < 1%.

For phosphoproteomic analysis, raw data were processed using Proteome Discoverer 3.0, and MS/MS spectra were searched against the reviewed SwissProt human proteome database. All searches were carried out with a precursor mass tolerance of 20 ppm at the MS level, considering charge states ranging from +2 to +7. Variable modifications included phosphorylation (S, T, Y) (+79.9949 Da), oxidation (Met) (+15.9949 Da), acetylation (protein N-terminus) (+42.0106 Da), while carbamidomethylation (Cys) (+57.0215 Da) was applied as a fixed modification during the database search. Additionally, two missed cleavages by trypsin were permitted. Fragment ions were allowed a mass deviation of 0.02 Da for the HCD data. Only peptides with at least 7 amino acids in length were considered. Peptide and protein identifications were filtered to maintain a false discovery rate (FDR) < 1%.

### 2.9. Plasmid Construction, Amplification and Transfection

For the overexpression of β-Catenin, the coding sequence was synthesized and inserted into the pCDH vector. The coding sequence of β-Catenin-S552A mutant was amplified using β-Catenin cDNA as a template and subsequently inserted into the pCDH vector. For virus preparation, the packaging vectors pMDL, VSVG, and REV were co-transfected with a control (pCDH empty vector) or β-Catenin/β-Catenin-S552A into HEK293T cells. To knock down FN1 and LOX, shRNA targeting FN1 or LOX was designed (Table S1) and cloned into pLKO.1-TRC vector. For virus production, the packaging vectors pMDL, VSVG, and REV were co-transfected with either shCTL or shLOX/shFN1 into HEK293T cells. After culturing for 48 or 72 h, the virus supernatants were collected. For cell transfection, U2OS cells were incubated with the virus in the presence of 10 μg/mL polybrene, followed by centrifugation for 30 min at 1500× *g* at 37 °C. The cells were used for further experiments 48 h post-transfection.

### 2.10. Statistical Analysis

The data were presented as mean ± standard deviation. A *t*-test was employed to compare mean values between the two groups, while a one-way analysis of variance was used to compare the mean values between multiple groups. *p* < 0.05 was considered statistically significant.

## 3. Results

### 3.1. HMF Exposure Promotes the Proliferation and Migration of U2OS Cells

**Figure 1:**
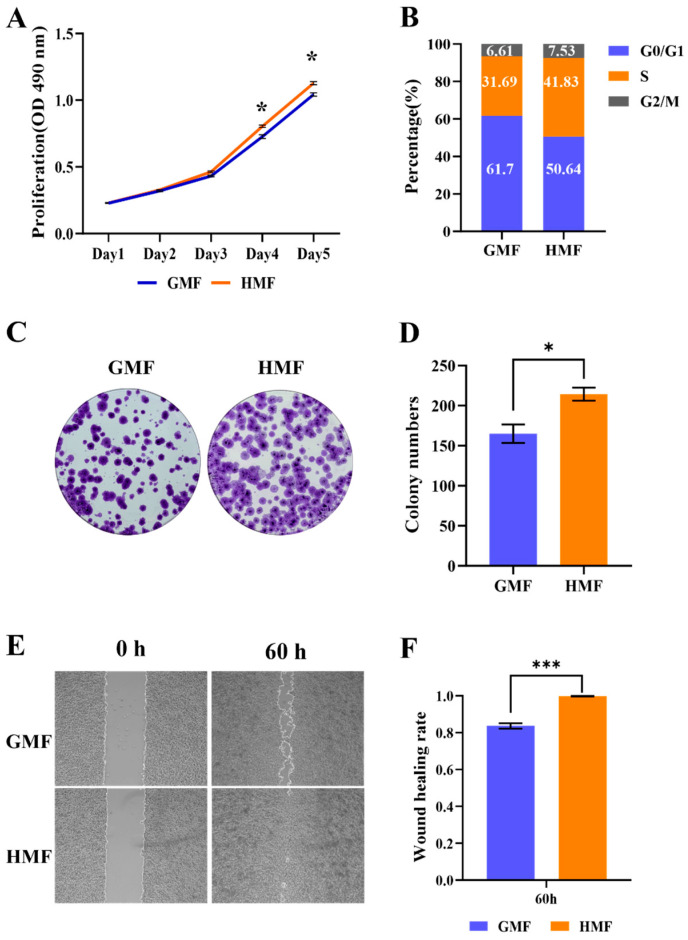
The HMF promotes U2OS cell proliferation and migration. (**A**) The MTS assay for assessing the proliferation of U2OS cells cultured in the GMF and the HMF (n = 4). (**B**) The cell cycle analysis of U2OS cells cultured in the GMF and the HMF. (**C**) The clone formation of U2OS cells cultured in the GMF and the HMF for 2 weeks (n = 3). (**D**) The quantification of clone numbers. (**E**) The wound healing assay for analyzing the migration of U2OS cells exposed to the GMF and the HMF (n = 9). (**F**) The migration efficiency of the cells in the HMF environment was significantly higher than that of the GMF controls. * *p* < 0.05, *** *p* < 0.001.

In our study, we initially validated the effect of HMF exposure on the proliferation of U2OS cells. As shown in the MTS assay results in Figure 1A, we observed that the HMF conditions significantly increased U2OS cell proliferation compared to the GMF group. Cell counting also indicated that the cell densities in the HMF group were higher than those in the GMF group after 3 days of culture (Figure S2). Cell cycle analysis revealed that the proportion of U2OS cells in the G0/G1 phase decreased, while the percentage of cells in the S phase increased (Figure 1B). Additionally, we further investigated the effects of long-term HMF exposure through a clonal formation study. As demonstrated in Figure 1C,D, clones in the HMF environment were larger, and the number of clones was greater compared to the GMF group. Subsequently, we evaluated the impact of HMF on cell migration using a wound healing assay. As indicated in Figure 1E, HMF exposure induced nearly complete closure of the pseudo-wound field produced in the cell monolayer after 60 h. The wound closure percentage was also quantified, as shown in Figure 1F, revealing that HMF exposure led to higher wound healing rates compared to the GMF group. Collectively, these results indicate that the HMF accelerates the proliferation and migration of U2OS cells.

### 3.2. HMF Exposure Induces Changes in Protein Phosphorylation Modifications

**Figure 2:**
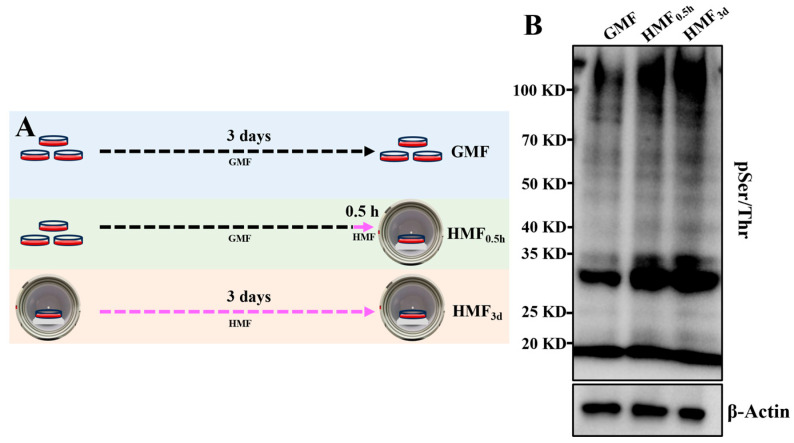
The HMF modulation of protein phosphorylation in U2OS. (**A**) The experimental timeline for U2OS cell exposure to the HMF. (**B**) WB analysis of global protein phosphorylation (pSer/Thr) in U2OS exposed to the HMF for 0.5 h and 3 days.

To investigate whether the HMF stimulates changes in protein phosphorylation, cells were cultured in the GMF or the HMF (Figure 2A), and global protein phosphorylation modifications were assessed using Western blot (WB). As shown in Figure 2B, the phosphorylated modifications in U2OS cells changed significantly at a macroscopic level when exposed to the HMF, even with a shorter exposure time of 0.5 h. This indicates that the U2OS cells can rapidly sense changes in magnetic fields, leading to alterations in protein phosphorylation levels within the cell. As the exposure duration of U2OS cells to the HMF was increased to 3 days, the extent of macroscopic changes in protein phosphorylation modifications became more pronounced (Figure 2B). These results indicate that HMF exposure leads to significant changes in protein phosphorylation within U2OS cells, suggesting that protein phosphorylation modifications serve as a significant regulatory mechanism through which cells respond to the HMF.

### 3.3. Quantitative Profiling of the HMF-Induced Phosphoproteome in U2OS Cells

**Figure 3:**
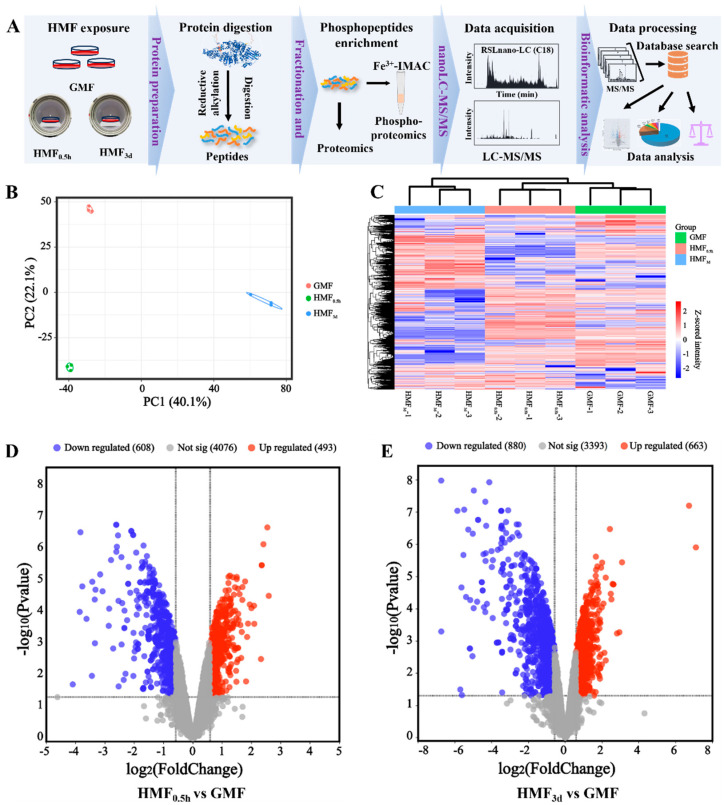
The phosphoproteomics for the identification of altered phosphosites and the proteins containing these sites induced by the HMF. (**A**) A workflow of proteomics and phosphoproteomics. (**B**) The principal component and hierarchical clustering analysis of the phosphorylated modifications identified in the GMF, HMF_0.5h_ and HMF_3d_ samples. (**C**) A global clustering heatmap displaying HMF-induced proteins with altered phosphosites. (**D**) Volcano plots illustrating the numbers of upregulated and downregulated phosphosites between the GMF and HMF_0.5h_. (**E**) Volcano plots illustrating the numbers of upregulated and downregulated phosphosites, as well as between the GMF and HMF_3d_.

Subsequently, a phosphoproteomics study was conducted to identify the altered phosphosites and the proteins harboring these sites induced by the HMF (Figure 3A). The cells were subjected to short-term (0.5 h) and long-term (3 days) exposure to the HMF, with U2OS cells cultured in the GMF serving as a control (Figure 2A). As a result, a total of 6546 phosphosites were identified from 2749 proteins (Figure S3). Principal component analysis and hierarchical cluster analysis indicated that samples from each group could be distinctly clustered, with the HMF-exposed groups clearly distinguished from the GMF group (Figure 3B). Furthermore, heatmap analysis also revealed significant differences in protein phosphorylation modifications between the HMF and GMF groups, as well as notable distinctions between the 3-day HMF exposure group and the 0.5 h exposure group (Figure 3C).

The result of quantitative analysis indicates that there are significant alterations (|FC| ≥ 1.5, *p* ≤ 0.05) in the phosphorylation modification levels of 1101 modification sites in the HMF exposure group after 0.5 h. This includes 608 sites with reduced modification levels and 493 sites with increased modification levels (Figure 3D), demonstrating a significant difference in the phosphorylation levels of proteins induced by the HMF. As the duration of exposure to the HMF increases, the number of sites experiencing differential phosphorylation also rises. After 3 days of exposure to the HMF environment, the phosphorylation levels of 1543 sites are altered. Among these, 663 sites exhibit an increased phosphorylation level, while 880 sites demonstrate a decreased phosphorylation level (Figure 3E). The number of distinct phosphorylation sites after 3 days of HMF exposure is significantly greater than that observed after just 0.5 h. This finding indicates a time-dependent pattern in the phosphorylation modifications, with an increase in the number of sites correlating with the duration of HMF exposure.

### 3.4. Phosphoproteomics Function Enrichment Analysis and Kinase Prediction

The distribution and subcellular localization of proteins within cells are crucial to their functions. Therefore, we analyzed the cellular distribution and subcellular localization of HMF-induced proteins with altered phosphosites (HMF_0.5h_ and HMF_3d_). Our findings indicate that those phosphoproteins are distributed across various subcellular compartments, including the cytosol, plasma membrane, nucleus (nucleoplasm, nuclear speckles and the nucleoli), and other organelles (such as the Golgi apparatus, vesicles, mitochondria, endoplasmic reticulum) (Figure S4). This suggests that the phosphorylation modification of the HMF-induced proteins is widespread in U2OS cells and may be involved in many life-regulating processes. Additionally, gene ontology (GO) biological process enrichment analysis revealed significant functional involvement in the regulation of the mitotic cell cycle process, cell cycle process and positive regulation of the cell cycle, all of which are related to cell cycle changes induced by HMF exposure. Other enriched biological processes include mRNA processing, DNA metabolic processing, chromatin organization, epigenetic regulation of gene expression and cytoskeleton regulation (Figure S5). The protein molecular function enrichment analysis predominantly highlighted cadherin binding, actin binding, chromatin binding, histone binding, transcription coregulator activity, protein domain binding, and ATP-dependent activity (Figure S5). Furthermore, cellular component analysis revealed that these phosphorylated proteins are components of the nuclear speck, chromosomal region, centrosome, spindle, nuclear envelope and periphery, spliceosomal complex, microtubule and actin cytoskeleton (Figure S5). Those results indicate that HMF-induced phosphorylation might play an important role in regulating specific electrostatic protein–protein and protein–nucleic acid interactions, thereby regulating cell proliferation and migration.

**Figure 4:**
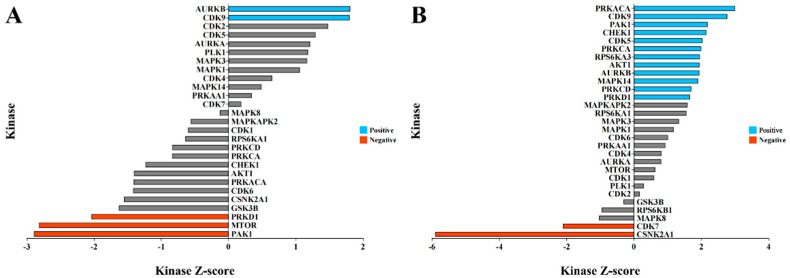
The potential kinase prediction analysis through KSEA of the HMF-regulated phosphosites suggests the involvement of several classes of kinases (substrate > 5). (**A**) The prediction of kinases associated with proteins exhibiting altered phosphosites after exposure to the HMF for 0.5 h. (**B**) The prediction of kinases associated with proteins exhibiting altered phosphosites after exposure to the HMF for 3 days. A Z-score > 0 indicates kinase activation by the HMF, whereas a Z-score < 0 indicates kinase inactivation by the HMF.Grey color indicates *p* > 0.05.

We further analyzed our phosphoproteomics dataset to predict the potential kinases responsible for these HMF-induced phosphorylation events through kinase–substrate enrichment analysis (KSEA). The results indicate that the HMF-induced phosphorylation events are associated with numerous kinases. Notably, exposure to the HMF for 0.5 h predicts a significant activation of AURKB and CDK9 (*p* < 0.05), while the activities of the kinases PRKD1, MTOR, and PAK1 are predicted to be inhibited (*p* < 0.05) (Figure 4A). The number of kinases regulating these differential phosphorylation modifications after 3 days of HMF exposure also increased. In addition to AURKB and CDK9, the kinases PAK1, CHEK1, CDK5, PRKCA, RPS6KA3, AKT1, MAPK14, PRKCD, and PRKD1 are predicted to be active (*p* < 0.05), while CDK7 and CSNK2A1 were significantly inhibited (*p* < 0.05) (Figure 4B). We hypothesize that when U2OS cells perceive changes in magnetic field strength from the GMF to the HMF, this leads to alterations in the activity of various kinases within the cell, thereby regulating changes in protein phosphorylation modifications. Consequently, these modifications control the transcription of relevant genes.

### 3.5. HMF Exposure Promotes the Phosphorylation of β-Catenin at S552

**Figure 5:**
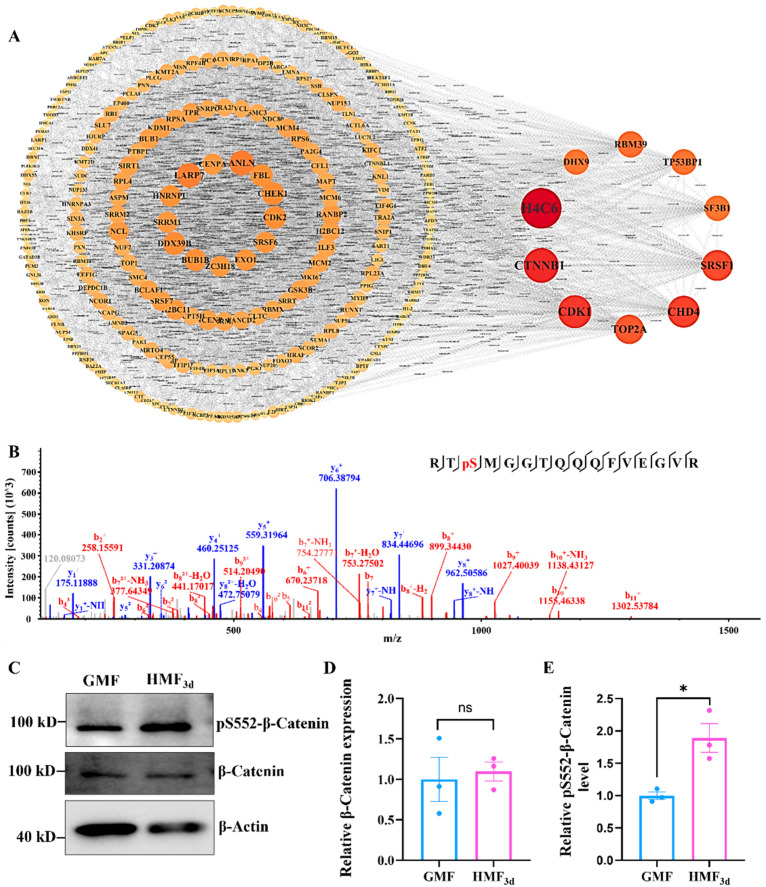
The HMF promotes the phosphorylation of β-Catenin at S552. (**A**) The PPI network derived from the total gene lists (HMF_3d_-induced proteins with altered phosphosites) and nodes with a degree greater than 10 were visualized. (**B**) The LC-MS/MS spectrum of the β-Catenin tryptic fragment Arg550-Arg565 indicates phosphorylation at S552. The detected b-ions and y-ions are indicated in red and blue, respectively. (**C**) The expression and phosphorylation of β-Catenin in U2OS cells, which were cultured in the GMF and HMF environments for 3 days, respectively (n = 3). (**D**,**E**) The relative expression levels of β-Catenin and its phosphorylation at S552 were quantified using ImageJ 1.52 software. * *p* < 0.05, ns indicates not significant, *p* > 0.05.

To systematically analyze and interpret the interactions among proteins with differential phosphorylation sites and elucidate the underlying regulatory mechanisms, we constructed the protein–protein interaction (PPI) network to identify hub phosphorylated proteins. Based on node degree, we selected and visualized the top 10 hub node proteins. Notably, the protein β-Catenin consistently occupies a pivotal position among the core proteins in U2OS cells exposed to the HMF environment for both 0.5 h and 3 days (Figure 5A and Figure S6). This suggests that β-Catenin likely plays a central role in the overall phosphorylation response network. The quantification of phosphoproteomic results reveals that in U2OS cells exposed to the HMF environment, the level of serine phosphorylation at position 552 of the β-Catenin significantly increases after both 0.5 h and 3 days of exposure (Figure 5B and Table S2). This suggests that phosphorylation at S552 may play a regulatory role in the HMF response process. To further validate these findings from the phosphoproteomic analysis, we employed WB to assess the expression levels of the β-Catenin in U2OS cells treated with the HMF, along with the levels of its S552 phosphorylation. The WB results demonstrated a significant enhancement in the pS552-β-Catenin phosphorylation level following HMF exposure (Figure 5C,E). Notably, there was no observable change in the β-Catenin expression levels after HMF exposure (Figure 5C,D), which aligns with the findings from both the quantitative proteomics and phosphoproteomics. Collectively, these findings suggest that HMF exposure promotes the phosphorylation of β-Catenin at the site of S552.

### 3.6. Phosphorylation of β-Catenin at S552 Promotes U2OS Cell Proliferation and Migration

**Figure 6:**
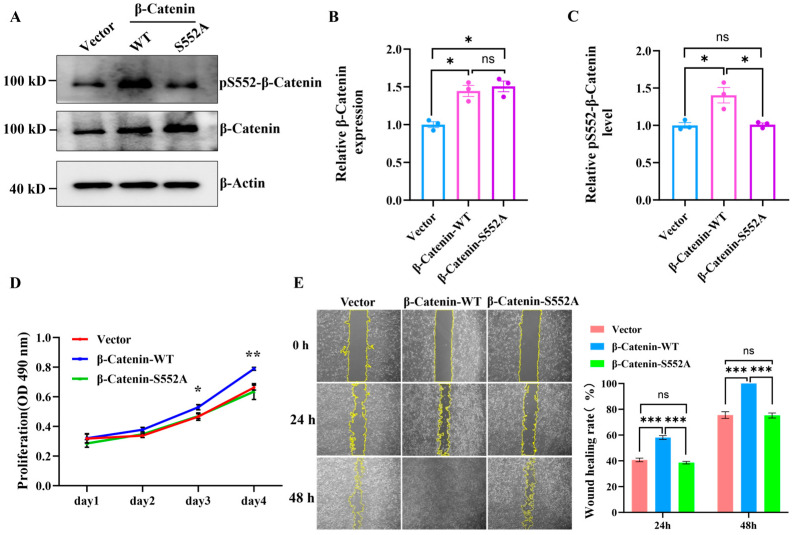
The phosphorylation of β-Catenin at the S552 site promotes U2OS cell proliferation and migration. (**A**) The WB analysis of β-Catenin (or β-Catenin-S552A) expression and phosphorylation levels in U2OS cells following 48 h of lentiviral infection (n = 3). (**B**,**C**) The quantification of relative β-Catenin expression and phosphorylation levels in U2OS cells. (**D**) The cell proliferation assay by MTS (n = 4). (**E**) The wound healing assay validating the effects of overexpression of β-Catenin-WT and β-Catenin-S552A on U2OS cell migration (n = 9). * *p* < 0.05, ** *p* < 0.01, and *** *p* < 0.001, ns indicates not significant, *p* > 0.05.

β-Catenin is a substrate protein of the kinase AKT. Previous studies have reported that β-catenin can be phosphorylated by AKT at S552 [26,27]. To further investigate the functional roles of the phosphorylation of the β-Catenin protein at S552, we performed mutagenesis at the serine site 552. We constructed both a wild-type β-Catenin (β-Catenin-WT) plasmid and a β-Catenin-S552A mutant plasmid. Following lentiviral packaging and subsequent infection of U2OS cells, the expression of wild-type β-Catenin-WT and the β-Catenin-S552A mutant was successfully induced within these cells (Figure 6A). The WB indicated that the expression levels of the β-Catenin protein in U2OS cells infected with the β-Catenin-WT and β-Catenin-S552A mutant viruses were significantly higher compared to the control group (Vector). Furthermore, there was no significant difference in the expression levels of the β-Catenin protein between the overexpression groups of β-Catenin-WT and β-Catenin-S552A (Figure 6B). Notably, the phosphorylation level of S552 in the β-Catenin-WT overexpression group is significantly elevated compared to both the control group (Vector) and the β-Catenin-S552A group (Figure 6C). Then, we confirmed the impact of S552 phosphorylation on the proliferation of U2OS cells. The results, illustrated in Figure 6D and Figure S7, indicated that the overexpression of β-Catenin-S552A does not affect U2OS cell proliferation. In contrast, U2OS cells overexpressing β-Catenin-WT exhibited a significantly faster proliferation rate compared to both the control group (Vector) and the β-Catenin-S552A overexpressing group. Subsequently, we found that phosphorylation at the S552 site influences the migration of U2OS cells. The results of the wound healing assays, illustrated in Figure 6E, revealed that the wound healing rate of U2OS cells overexpressing β-Catenin-WT was significantly higher than that of the control group (Vector) and those overexpressing β-Catenin-S552A. In contrast, the overexpression of β-Catenin-S552A yielded wound healing rates comparable to those of the control group, with no significant difference observed. Furthermore, a phosphorylation-mimic β-catenin-S552D mutant was overexpressed in U2OS cells (Figure S8A,B). In contrast to the expression of β-catenin-WT, the expression of β-catenin-S552D significantly enhanced U2OS cell proliferation (Figure S8C,D) and migration (Figure S8E,F). These results indicate that HMF exposure induces β-catenin phosphorylation at S552, thereby promoting U2OS cell proliferation and migration.

### 3.7. HMF Exposure Upregulates the Expression of LOX and FN1

**Figure 7:**
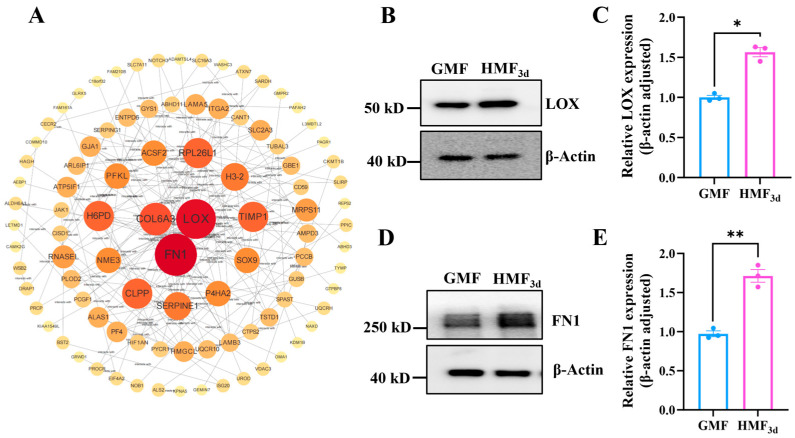
HMF exposure modulated the expression of LOX and FN1. (**A**) The PPI network derived from the total gene lists of the HMF_3d_-induced differentially expressed proteins. (**B**,**C**) The relative expression levels of LOX (n = 3). (**D**,**E**) The relative expression levels of FN1 (n = 3). * *p* < 0.05, ** *p* < 0.01.

Studies have reported that the phosphorylation of β-catenin at S552 by AKT contributes to β-catenin transcriptional activity [26,27,28,29]. Phosphorylation of β-catenin at S552 facilitates the disassociation of β-Catenin with cadherin and promotes its translocation to the nucleus [30,31]. Nuclear-localized β-catenin interacts with transcription factors, leading to the increased expression of downstream genes, which in turn enhances tumor cell invasion and proliferation. Therefore, we further analyzed the results of the quantitative proteomics. A total of 7166 proteins were identified (Figure S9A). Principal component analysis and hierarchical cluster analysis showed that HMF-exposed groups were clearly distinguished from the GMF group (Figure S9B). Heatmap analysis revealed significant differences in protein expression between the HMF_3d_ and GMF groups (Figure S9C). Compared to the GMF, U2OS cells exposed to the HMF for 3 days exhibited significant changes (|FC| ≥ 1.5, *p* ≤ 0.05) in expression levels for 391 proteins, including 218 upregulated and 173 downregulated proteins (Figure S9D). Notably, the PPI network demonstrated that LOX and FN1 are highly connected nodes (Figure 7A), which were upregulated in the HMF_3d_ group (Table S3).

To further validate the impact of HMF exposure on the expression of LOX and FN1, we first employed qPCR to assess the mRNA levels of their respective coding genes. As illustrated in Figure S10, the mRNA levels of LOX and FN1 remained unchanged after 0.5 h of HMF exposure. However, a significant upregulation in the mRNA expression of these genes was observed after 3 days of exposure, which is consistent with the findings from the quantitative proteomics analysis. Additionally, the WB results (Figure 7B,D) also show that a 3-day exposure to the HMF significantly increases the protein expression levels of LOX and FN1 compared to the GMF group (Figure 7C,D). These findings suggest that the HMF exposure enhances the transcription of *FN1* and *LOX*, resulting in a notable upregulation of their expression.

### 3.8. FN1 and LOX Promote U2OS Cell Proliferation and Migration

**Figure 8:**
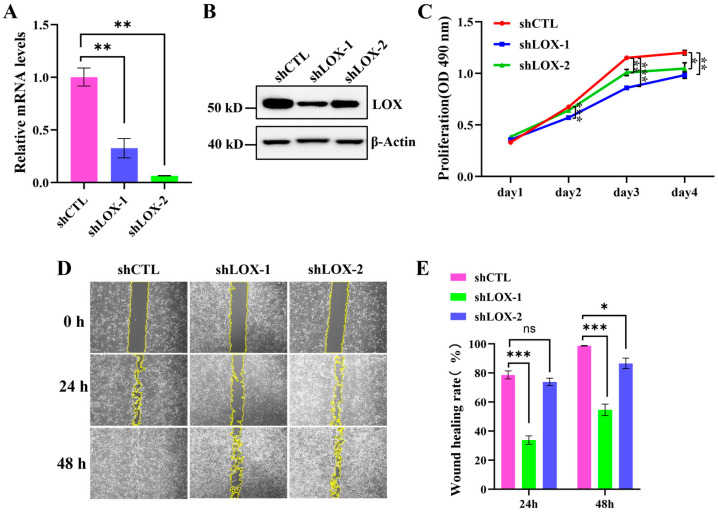
The LOX knockdown inhibits the proliferation and migration of U2OS. (**A**) The relative LOX mRNA levels in U2OS cells infected with shCTL and shLOX for 48 h (n = 3). (**B**) The WB analysis of the LOX expression in U2OS cells infected with shCTL and shLOX. (**C**) The MTS assay evaluating cell proliferation (n = 4). (**D**,**E**) The wound healing assay validating the effects of the LOX knockdown on U2OS cell migration (n = 9). * *p* < 0.05, ** *p* < 0.01, and *** *p* < 0.001, ns indicates not significant, *p* > 0.05.

LOX is a copper-dependent lysyl oxidase that primarily contributes to the assembly of the extracellular matrix. It plays crucial roles in cellular communication, fate determination, differentiation, and developmental processes. Recent studies have indicated that LOX is involved in tumor suppression and metastasis, as well as in regulating tumor cell proliferation and various other cellular activities [32,33]. To investigate the functions of LOX, we constructed shRNAs to knock down LOX. Our findings indicate that the shRNA treatment effectively reduces the mRNA levels of LOX in U2OS cells (Figure 8A). Furthermore, the WB results confirm that the expression of the LOX protein is significantly decreased (Figure 8B). We then validated the effect of the LOX knockdown on the proliferation and migration of U2OS cells. The MTS assay and cell count results revealed that the LOX knockdown significantly inhibits the proliferation of U2OS cells both in the GMF (Figure 8C and Figure S11) and the HMF (Figure S12). Additionally, the wound healing assay results demonstrated that the rate of wound healing in the LOX knockdown group was significantly slower than that in the shCTL group, both in the GMF (Figure 8D,E) and the HMF (Figure S13A), indicating that LOX reduction also impairs the migratory capacity of the cells.

**Figure 9:**
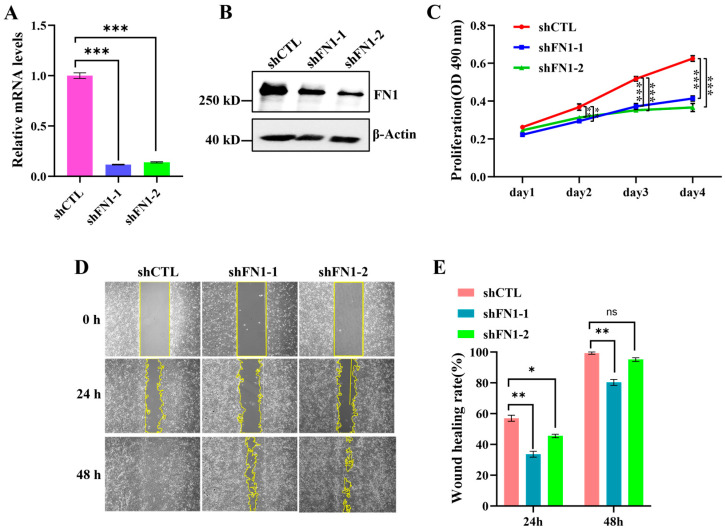
FN1 knockdown inhibits the proliferation and migration of U2OS. (**A**) The relative FN1 mRNA levels in U2OS cells infected with shCTL and shFN1 for 48 h (n = 3). (**B**) The WB analysis of FN1 expression in U2OS cells infected with shCTL and shFN1. (**C**) The MTS assay evaluating cell proliferation (n = 4). (**D**,**E**) The wound healing assay validating the effects of the FN1 knockdown on U2OS cell migration (n = 9). * *p* < 0.05, ** *p* < 0.01, and *** *p* < 0.001, ns indicates not significant, *p* > 0.05.

FN1 (fibronectin 1) is a glycoprotein that directly participates in the formation of the extracellular matrix. This protein is widely expressed across various cell types and has been shown to play significant roles in processes such as adhesion, proliferation, migration, and invasion of cancer cells [34,35]. To further investigate the functions of FN1, we knocked down FN1 to assess its effects on the proliferation and migration of U2OS cells. The treatment with shRNA effectively reduced the mRNA levels of FN1 (Figure 9A) and led to a significant decrease in FN1 protein expression (Figure 9B). We further validated the effect of FN1 knockdown on the proliferation and migration of U2OS cells. The results indicated that FN1 knockdown significantly inhibits the proliferation of U2OS cells in both the GMF (Figure 9C and Figure S11) and the HMF (Figure S12B–D). Additionally, the wound healing assay results showed that the rate of wound healing in the FN1 knockdown group was significantly slower than that in the shCTL group, both in the GMF (Figure 9D,E) and the HMF (Figure S13B), indicating that FN1 reduction also inhibits the migratory ability of cells. In summary, the knockdown of either LOX or FN1 effectively inhibits the proliferation and migration of U2OS cells, suggesting that LOX and FN1 play a role in promoting these processes.

## 4. Discussion

Protein phosphorylation is an important regulatory mechanism by which cells respond to environmental changes. The GMF is an important environmental factor; a significant reduction in magnetic field strength inevitably leads to some degree of impact on cells. In this study, our research findings reveal that U2OS cells exhibit macroscopic changes in protein phosphorylation levels within 0.5 h of exposure to the HMF. Through quantitative proteomics for phosphorylation, we identified over 1000 distinct phosphorylation sites. This indicates the rapid capability of U2OS cells to perceive variations in magnetic field strength, leading to changes in protein phosphorylation. This suggests that the U2OS cells have the capability to swiftly respond to the alterations in magnetic field strength, thereby triggering changes in protein phosphorylation. Moreover, as the exposure time to the HMF increases, the differential protein phosphorylation modifications also incrementally grow, indicating that protein phosphorylation is an important regulatory mechanism for cells to adapt to the HMF environment. These results demonstrate the HMF’s modulation of protein phosphorylation within cells.

The localization and distribution of proteins within a cell often determine their function. In U2OS cells, HMF-induced phosphorylation-altered proteins exhibit a broad intracellular distribution, with notable enrichment in the nucleus. These nuclear locations include the nucleoplasm, nucleoli, nuclear speckles, nuclear bodies, nuclear membrane, centrosome, and nucleoli fibrillar centers. The GO biological process enrichment analysis reveals that proteins are significantly associated with mRNA processing, RNA localization, regulation of DNA metabolic processes, chromosome organization and segregation, DNA-templated transcription, and nuclear organization. The enrichment analysis of cellular components indicates that these proteins are localized to nuclear speckles, centrosomes, chromosome regions, the nuclear periphery, spliceosomal complexes, the nuclear envelope, nuclear chromosomes, transcription regulator complexes, and the spindle. Furthermore, molecular function analysis shows that these proteins are enriched in chromatin binding, transcription coregulator activity, histone modification activity, histone binding, mRNA binding, and promoter-specific chromatin binding. Additionally, the top 10 hub proteins in the PPI network include proteins associated with RNA splicing (such as SRRM1, SRSF1, SF3B1, U2AF2, HNRNPC, etc.) and transcription factors or proteins that regulate gene transcription (such as BRD4, MYC, RBM39, CTNNB1, CHD4, etc.). This comprehensive analysis suggests that rapid changes in protein phosphorylation in response to HMF sensing likely initiate the transcription of relevant genes, enabling cells to adapt to variations in magnetic field strength.

HMF-induced phosphorylation events are associated with numerous kinases. As the exposure time to the HMF increases, the number of distinct phosphorylation sites influenced by the HMF significantly rises, corresponding to an increase in kinases that regulate these differential phosphorylation modifications. In proteomics, more than 260 protein kinases were identified. However, quantitative analysis revealed that exposure to the HMF for 3 days did not lead to changes in the expression levels of these kinases, suggesting that the observed changes in phosphorylation levels may be attributed to alterations in enzyme activity rather than changes in expression. Furthermore, phosphoproteomics results revealed the presence of phosphorylation modifications in over 120 kinases, of which 53 kinases exhibited significant alterations. In our previous study, we found that neural stem cells exposed to the HMF environment for 15 min exhibited a reduced phosphorylation of GSK-3β at S9 [36], which would activate GSK-3β. This suggests that the HMF can regulate kinase activity through the modulation of phosphorylation modifications on related kinases, thereby influencing the phosphorylation modifications of proteins within the cell.

β-Catenin, a crucial component of both WNT signaling and cell–cell adhesion, plays a central role in cell proliferation, differentiation, polarity, morphogenesis, and development. The localization of β-Catenin in different cellular compartments leads to the formation of distinct complexes, which execute specific cellular functions. Previous studies have demonstrated that β-Catenin can be phosphorylated by AKT at S552 [27,30,37]. In this study, the HMF exposure enhances phosphorylation of β-catenin at S552, which promotes the proliferation and migration of U2OS cells. The phosphorylation status of β-Catenin affects its distribution within the cell. Specifically, the phosphorylation of β-catenin at S552 by AKT enhances the association between β-Catenin and 14-3-3ζ, facilitating the disassociation of β-Catenin from cell–cell contacts and its subsequent translocation to the cytosol and nucleus [27,38]. Upon translocation into the nucleus, β-Catenin directly interacts with transcription factors of the TCF/LEF-1 family, leading to the increased expression of downstream genes that promote tumor cell growth and invasion [39,40]. Furthermore, quantitative proteomics reveals that a 3-day exposure to the HMF upregulates the transcription and translation of the LOX and FN1 genes within U2OS cells, while a 0.5 h exposure was not. The knockdown of LOX or FN1 significantly inhibits the proliferation and migration of U2OS cells. We hypothesize that HMF exposure enhances β-Catenin phosphorylation at S552, promoting its translocation to the nucleus and activation of LOX and FN1 transcription to enhance the proliferation and migration of U2OS cells.

## 5. Conclusions

In conclusion, we found that the HMF enhances the proliferation and migration of U2OS cells. The exposure of U2OS cells to the HMF environment resulted in significant alterations in intracellular protein phosphorylation. Notably, HMF exposure enhanced the phosphorylation of β-catenin at S552, and this increased phosphorylation promoted U2OS proliferation and migration. Furthermore, exposure to HMF upregulated the expression of LOX and FN1. The knockdown of either LOX or FN1 suppressed the proliferation and migration of the U2OS cells in both the GMF and HMF conditions. Collectively, these results indicate that the HMF environment facilitates the phosphorylation of β-catenin at S552 and the upregulation of expression of LOX, FN1, thereby promoting the proliferation and migration of U2OS cells.

## Data Availability

The original contributions presented in this study are included in the article/Supplementary Materials. Further inquiries can be directed to the corresponding authors.

## Supplementary Materials

The following supporting information can be downloaded at https://www.mdpi.com/article/10.3390/cells15080727/s1. Figure S1: Magnetic field intensity distribution within a permalloy magnetic shield bucket is illustrated. (A) Technical drawings of the hypomagnetic field device. (B) shows that the magnetic field intensity within the permalloy magnetic shield bucket is less than 50 nT; Figure S2: HMF promotes U2OS cell proliferation. (A) U2OS cells (1.1 × 10^4^ cells) were seeded in 6-cm cell culture dishes and cultured in HMF and GMF environments, respectively. (B) After 3 days of culture, cells were harvested, resuspended in 1 mL of DMEM medium, and enumerated using automatic cell counter (Countstar BioTech) (n = 3). * *p* < 0.05, ** *p* < 0.01, *** *p* < 0.001; Figure S3: The number of phosphorylated sites identified in each group; Figure S4: Localization and distribution of these HMF-induced proteins with altered phosphosites within U2OS cells; Figure S5: The biological processes (BP), cellular components (CC), and molecular functions (MF) of HMF-induced phosphorylated proteins using Metascape; Figure S6: The protein-protein interaction network derived from the total gene lists (HMF_0.5h_-induced proteins with altered phosphosites) and the top 10 hub proteins is presented. Nodes with a degree greater than 10 were visualized using Cytoscape. The degree of a node is defined as the number of edges connected to it. A node with a high degree signifies a hub with numerous neighboring nodes; Figure S7: Cell proliferation was assessed through cell counting. U2OS cells (2 × 10^4^ cells) were seeded in 6-cm cell culture dishes and cultured in GMF environments for 1 and 3 days, respectively. After 3 days of culture, the cells were harvested, resuspended in 1 mL of cell culture medium, and counted using an automatic cell counter (Countstar BioTech) (n = 3). * *p* < 0.05, ** *p* < 0.01, *** *p* < 0.001; Figure S8: Overexpression of β-Catenin-S552D enhances the proliferation and migration of U2OS cells. (A) WB analysis of β-Catenin and β-Catenin-S552D expression levels in U2OS cells following lentiviral infection (n = 3). (B) Quantification of relative β-Catenin expression levels in U2OS cells. (C) and (D) Cell proliferation assays conducted through cell counting (n = 3). (E) and (F) Wound healing assays demonstrating the effects of overexpression of β-Catenin-WT and β-Catenin-S552D on U2OS cell migration (n = 5). * *p* < 0.05, ** *p* < 0.01, *** *p* < 0.001; Figure S9: Proteomic analysis of U2OS cells cultured in GMF and U2OS cells exposed to a HMF for 0.5 h and 3 days. (A) The number of proteins identified in each group. (B) Principal component analysis and hierarchical clustering analysis of the proteins identified in GMF, HMF_0.5h_ and HMF_3d_ groups. (C) Heat map of z-scored protein abundances of the proteins differentially expressed through HMF exposure. (D) Volcano plots illustrating the numbers of upregulated and downregulated proteins between the GMF and HMF_3d_; Figure S10: HMF exposure for 3 days enhances the transcription of *LOX* and *FN1*. (A) Relative mRNA levels of LOX in U2OS cells exposed to HMF for 0.5 h and 3 days (n = 3). (B) Relative mRNA levels of FN1 in U2OS cells exposed to HMF for 0.5 h and 3 days (n = 3). * *p* < 0.05, ** *p* < 0.01, *** *p* < 0.001; Figure S11: FN1 or LOX knockdown inhibits the proliferation of U2OS cells under GMF conditions. (A) U2OS cells (1.1 × 10^4^ cells) were seeded in 6-cm cell culture dishes and cultured in GMF environments for 1 and 3 days, respectively. (B) After 3 days of culture, cells were harvested, resuspended in 1 mL of cell culture medium, and counted using an automatic cell counter (Countstar BioTech) (n = 3). * *p* < 0.05, ** *p* < 0.01, *** *p* < 0.001; Figure S12: FN1 or LOX knockdown inhibits the proliferation of U2OS cells under HMF conditions. (A) and (B) MTS assay assessing the proliferation of cells treated with shRNA in HMF (n = 4). (C) U2OS cells (1.1 × 10^4^ cells) were seeded in 6-cm cell culture dishes and cultured in HMF environments for 1 and 3 days, respectively. (D) After 3 days of culture, cells were harvested, resuspended in 1 mL of cell culture medium, and counted using an automatic cell counter (Countstar BioTech) (n = 3). * *p* < 0.05, ** *p* < 0.01; Figure S13: Knockdown of FN1 or LOX inhibits the migration of U2OS cells under HMF conditions. (A) Wound healing assay demonstrating the effects of LOX knockdown on U2OS cell migration in HMF (n = 5). (B) Wound healing assay demonstrating the effects of FN1 knockdown on U2OS cell migration in HMF (n = 5). * *p* < 0.05, ** *p* < 0.01, *** *p* < 0.001; Table S1: The sequences of shRNA control and shRNA targeting human *FN1* and *LOX*; Table S2: The relative level of serine phosphorylation at position 552 of the β-Catenin after both 0.5 h and 3 days of exposure in HMF compared to GMF; Table S3: Quantitative proteomic analysis of FN1 and LOX in U2OS cells cultured in HMF_3d_ compared to those cultured in GMF_3d_; Table S4: The sequences of qPCR primers.

## Abbreviations

The following abbreviations are used in this manuscript:

HMF	Hypomagnetic field
LOX	Protein-lysine 6-oxidase
FN1	Fibronectin 1
GMF	Geomagnetic field
ACN	Acetonitrile
PPI	Protein–protein interaction
LC-MS/MS	Liquid chromatography-mass spectrometry/mass spectrometry
KSEA	Kinase-substrate enrichment analysis
