# Supplementary material for: Hypomagnetic Field Enhances U2OS Cell Proliferation and Migration by Promoting β-Catenin Phosphorylation and Upregulating FN1 and LOX Expression
Source: Cells. 2026 Apr 19;15(8):727. doi: 10.3390/cells15080727 (PMC13115504; doi:10.3390/cells15080727)
Supplement: Supplementary file 1 [file cells-15-00727-s001.zip › cells-4148340-supplementary.pdf]

Supplementary Information

Hypomagnetic field enhances U2OS cell proliferation and migration by promoting β-Catenin phosphorylation and upregulating FN1 and LOX expression

Taotao Gao[1,2,†], Wenfeng Zhong[3,†], Mengli Tao[1,2], Yu Guo[1,2], Kun Yang[4], Yaohui He[5], Guosheng Hu[3], Long Li[1,2,*], Xiangyan Kong[4], Fulai Li[1,2,*], Yufen Zhao[1,2]

1 Institute of Drug Discovery Technology, Ningbo University, Ningbo 315211, Zhejiang, China

2 Qian Xuesen Collaborative Research Center of Astrochemistry and Space Life Sciences, Ningbo University, Ningbo 315211, Zhejiang, China

3 Biomedical Research Center of South China, College of Life Sciences, Fujian Normal University, Fuzhou, 350117, Fujian, China

4 Faculty of Electrical Engineering and Computer Science, Institute for Future Wireless Research (IFWR), Ningbo University, Ningbo, 315211, Zhejiang, China

5 MOE Key Lab of Rare Pediatric Diseases, Hengyang Medical School, University of South China, Hengyang, 421001, Hunan, China

* Corresponding authors: lilong@nbu.edu.cn; lifulai@nbu.edu.cn

† These authors contributed equally to this work.

1.1 Materials and regents

DMEM medium was purchased from Shanghai Zhongqiaoxinzhou Biotech. Fetal bovine serum (FBS) was bought from Biological Industries. The MTS assay kit was obtained from Promega. Methanol and crystal violet were all procured from Sangon Biotech. RNase, PhosSTOP phosphatase inhibitor cocktail, and protease inhibitors were obtained from Roche. PMSF was purchased from NCM Biotech. Both the BCA Protein Quantification Kit and the PAGE Color Rapid Gel Preparation Kit were purchased from Uelandy. Ultrafiltration centrifugal tubes were purchased from Merck Millipore. Tris(2-carboxyethyl) phosphine hydrochloride (TCEP), urea, and β-mercaptoethanol were purchased from Macklin. The Fe-NTA phosphopeptide enrichment kit was purchased from Thermo Fisher Scientific. Ammonium bicarbonate and iodoacetamide were purchased from Sigma. Desalting Sep-Pak C18 cartridges were purchased from Waters. The total RNA extraction kit was purchased from Omega. qPCR SYBR Green Master Mix, puromycin dihydrochloride solution, and Hieff Canace® Plus High-Fidelity DNA Polymerase were all purchased from Yeasen. mRNA reverse transcription reagents and plasmid extraction kits were both purchased from Vazyme.

Antibodies against β-actin (AC206, 1:50,000 dilution for WB), LOX (A11504, 1:1,000 dilution for WB), β-Catenin (A19657, 1:4,000 dilution for WB), LOX (A11504, 1:1,000 dilution for WB), phospho-β-catenin (AP1315, 1:2,000 dilution for WB) were all purchased from ABclonal. The fibronectin (FN1) recombinant rabbit monoclonal antibody was bought from HUABIO (JF0582, 1:1,000 dilution for WB). The anti-phosphoserine/threonine rabbit polyclonal antibody was acquired from ECM Biosciences (PP2551, 1:1,000 dilution for WB). Opti-MEM and MEM non-essential amino acids solution were both purchased from Gibco. The polybrane transfection reagent was bought from Merck. ECL chemiluminescent substrate and non-reducing 5×protein loading buffer were both purchased from Shanghai Epizyme. HRP-conjugated goat anti-rabbit IgG (H+L) was purchased from Beyotime.

1.2 Cell cycle analysis by flow cytometry

Cells grown in GMF and HMF for 3 days being washed with PBS, and then

detached from cell culture plate into single cell suspension. Washed twice with ice-cold PBS, the detached cells were fixed overnight in 2 mL of 70% ethanol at -20℃. Before staining, washing cells twice with cold PBS, and centrifuge at 4°C for 5min (3000×g) and remove supernatant completely, only leave cell pellet in the tube. The cell deposits were re-suspended in 0.5 mL of freshly prepared staining solution (0.1% Triton X-100, 0.2 mg/mL RNAse A, PI and incubate 37°C for 15 minutes in darkness. Subsequently, the cells were analyzed by flow cytometry (BECKMAN CytoFlex S).

1.3 RNA extraction and quantitative real-time PCR

Total RNA was isolated with the Total RNA Kit I (Omega) following the manufacturer's protocol. First-strand cDNA was synthesized from 1 µg of RNA using HiScript II SuperMix (Vazyme). Quantitative real-time PCR was subsequently carried out on a CFX Connect™ Real-Time PCR Detection System (Bio-Rad) with SYBR Green PCR Master Mix (Yeasen) according to the manufacturers' instructions. The sequences of primers used for qPCR reactions were listed in Table S4.

1.4 Western blotting

All adhesive cells were washed twice with cold PBS and subsequently ice-cold lysis buffer (50 mM Tris-HCl, 150 mM NaCl, 1% Triton-X-100, 1% SDC and 0.1% SDS) with 1 mM phenylmethanesulfonyl fluoride (PMSF) and 1× PhoSTOP was added and cells were scraped into a 1.5 mL centrifuge tube and incubated on ice for 10 min. Collected cells were ultrasonicated (5×3 seconds bursts) to disrupt cells and shear DNA, and lysates were clarified by centrifugation (14,000 g for 10 min at 4°C). And then BCA Protein Assay Kit was used to detect the protein concentration. For immunoblotting, the supernatant was directly boiled in SDS sample buffer and analyzed immediately using freshly prepared Bis-Tris polyacrylamide minigels with a 10% resolving gels. About 30 μg samples were resolved at 120 V for 1.5 hours. Proteins were transferred to immobilon FL PVDF membranes at 100 V for 1.5 hours at 4°C and immediately incubated for 60 min at RT in blocking buffer (5% BSA in TBST). Primary antibodies were diluted in TBST (containing 0.5% BSA), incubated with membranes overnight at 4 ℃. Membranes were washed four times for 15 min each with 0.1% TBST before incubation with secondary antibodies for 60 min at

room temperature. After incubation with secondary antibodies, membranes were washed least four times for 15 min each with TBST. After incubation with secondary antibodies, membranes were washed least four times for 15 min each with TBST. Finally, Immunoblots were imaged on a ChemiDocTM MP Imaging System. β-actin was used as an endogenous reference for quantitation. Quantitative analysis of WB images was conducted using ImageJ software. Images were captured with the same exposure time, and the optical density values of the target protein bands were measured using ImageJ to determine the relative intensity. Furthermore, the optical density values of the target protein in each sample were normalized by dividing them the corresponding values of internal reference protein (β-actin) to eliminate experimental variability and obtain the relative expression levels. Moreover, the WB images presented in the manuscript are representative of at least three independent experiments. These images reflect the trends in protein expression or phosphorylation levels across different experiments and are consistent with the quantitative data.

1.5 Bioinformatics analysis

The analysis of biological processes, cellular components, molecular functions (MF), and subcellular localization of HMF-induced phosphorylated proteins was performed using Metascape. The topological and functional enrichment analysis of the PPI network were generated by STRING and Cytoscape. Nodes with a degree greater than 10 were visualized using Cytoscape. Hub proteins in the PPI network represent highly connected nodes with specific biological properties, indicating their more important status within the network, where larger nodes, darker colors, and more densely connected lines indicate greater importance. Additionally, kinases were predicted through kinase-substrate enrichment analysis (KSEA).

2. Results

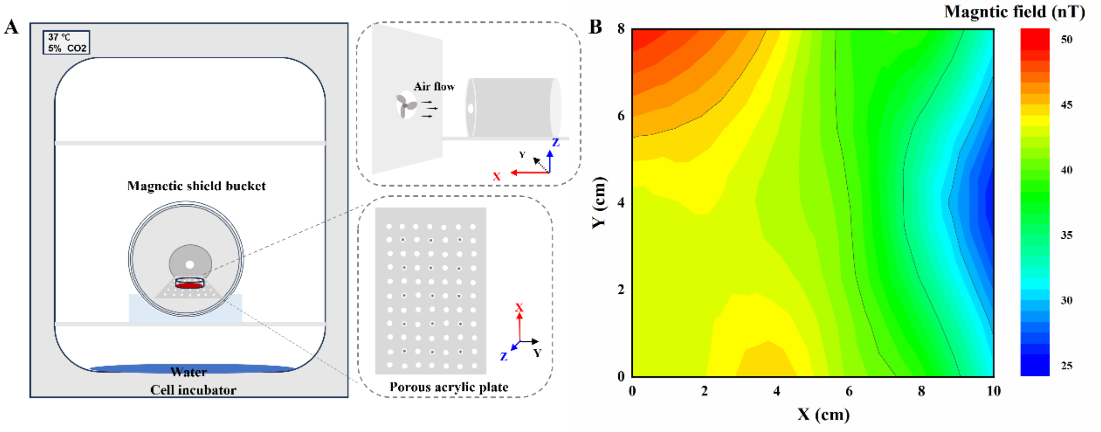

Figure S1. Magnetic field intensity distribution within a permalloy magnetic shield bucket is illustrated. (A) Technical drawings of the hypomagnetic field device. (B) shows that the magnetic field intensity within the permalloy magnetic shield bucket is less than 50 nT.

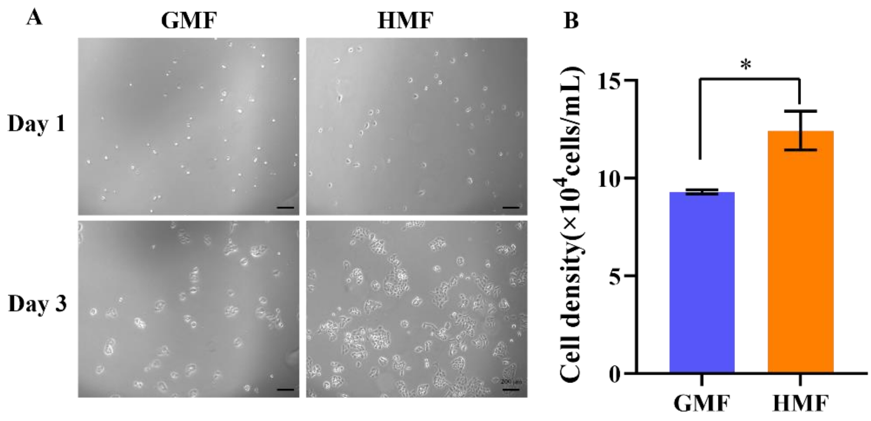

Figure S2. HMF promotes U2OS cell proliferation. (A) U2OS cells (1.1×10^4 cells) were seeded in 6-cm cell culture dishes and cultured in HMF and GMF environments, respectively. (B) After 3 days of culture, cells were harvested, resuspended in 1 mL of DMEM medium, and enumerated using automatic cell counter (Countstar BioTech) (n=3). *p<0.05, **p<0.01, ***p<0.001.

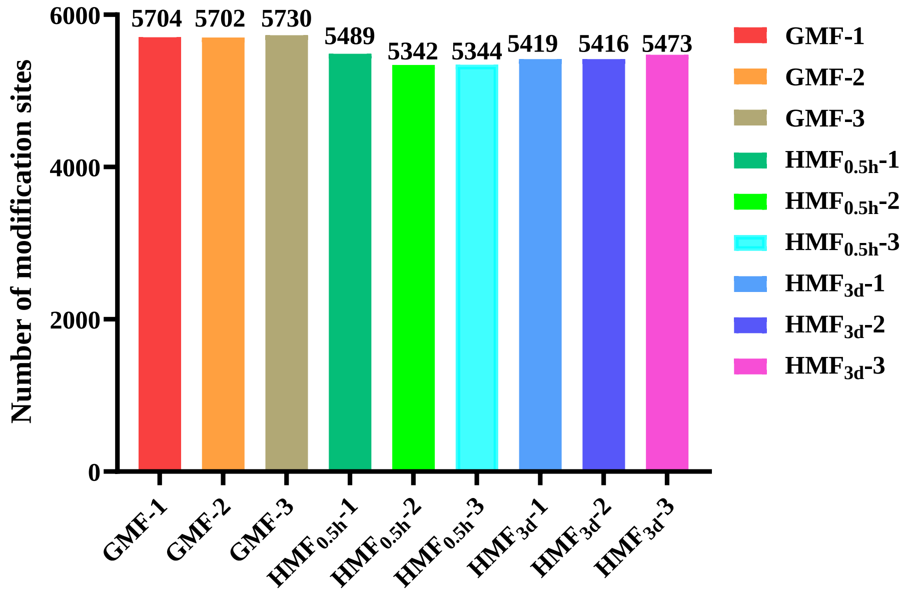

Figure S3. The number of phosphorylated sites identified in each group.

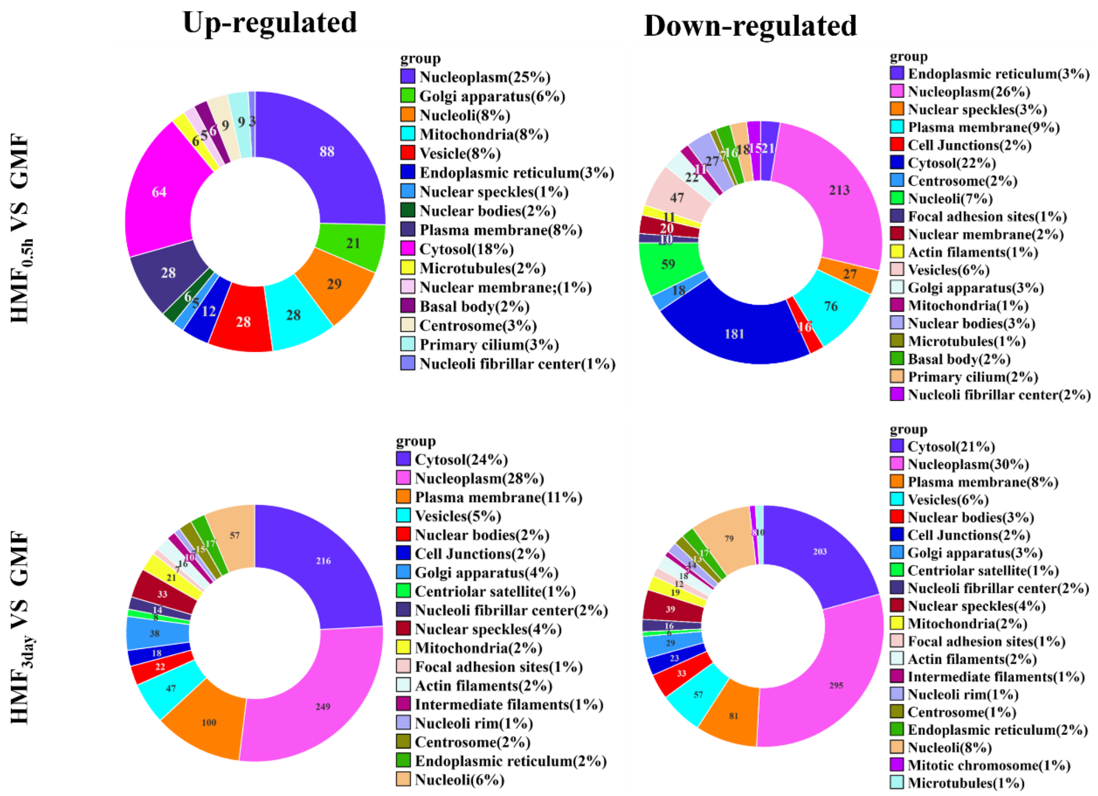

Figure S4. Localization and distribution of these HMF-induced proteins with altered phosphosites within U2OS cells.

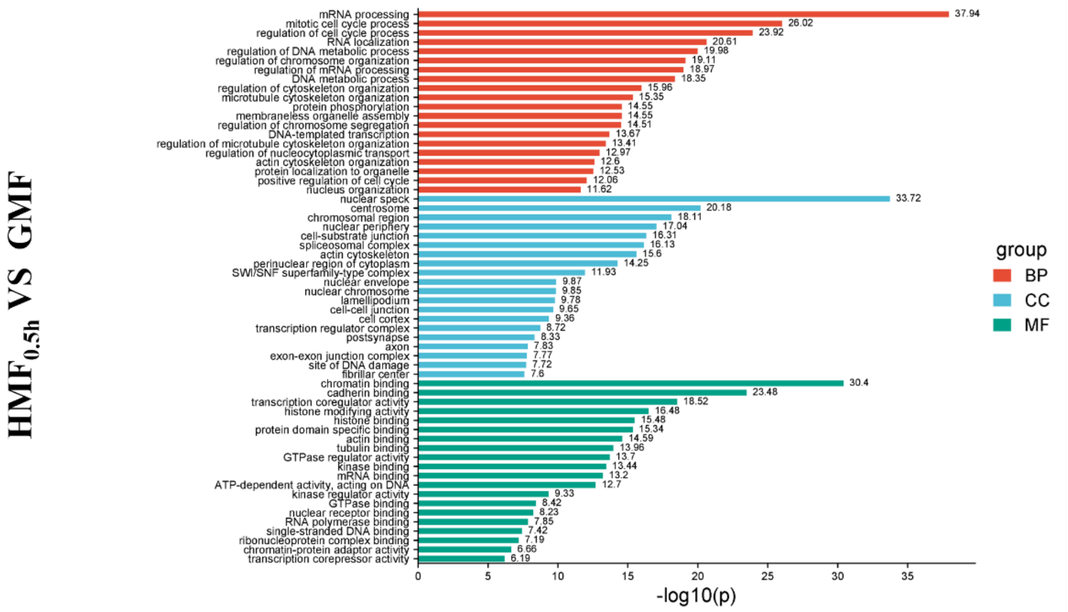

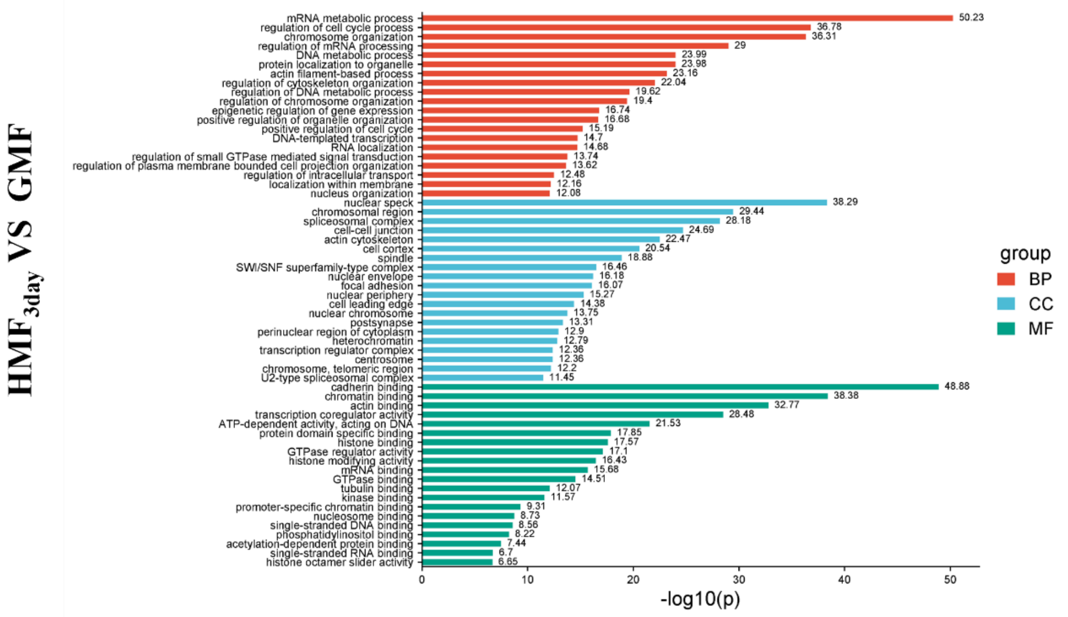

Figure S5. The biological processes (BP), cellular components (CC), and molecular functions (MF) of HMF-induced phosphorylated proteins using Metascape.

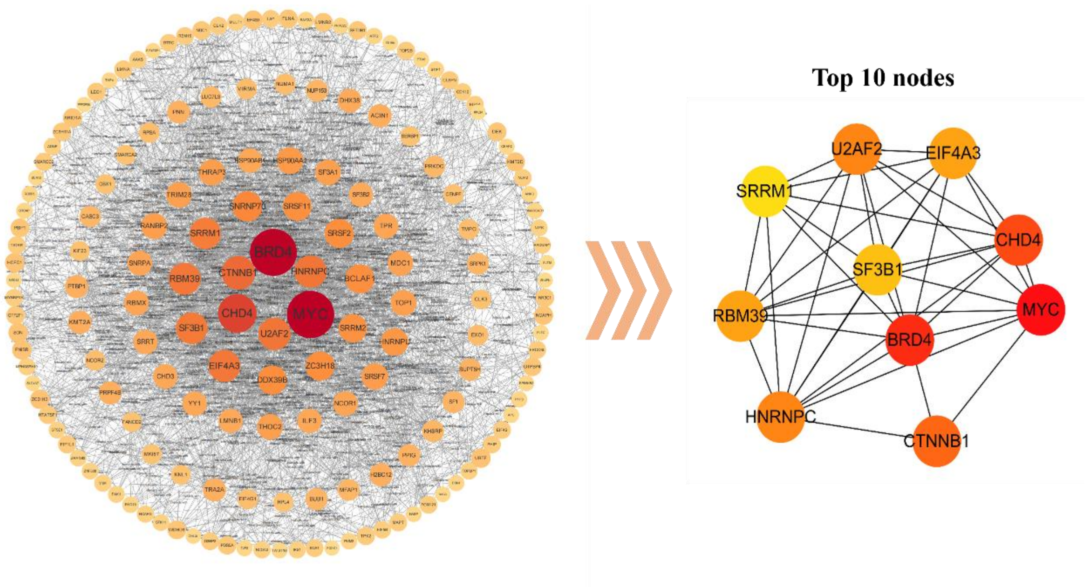

Figure S6. The protein-protein interaction network derived from the total gene lists ($HMF_{0.5h}$-induced proteins with altered phosphosites) and the top 10 hub proteins is presented. Nodes with a degree greater than 10 were visualized using Cytoscape. The degree of a node is defined as the number of edges connected to it. A node with a high degree signifies a hub with numerous neighboring nodes.

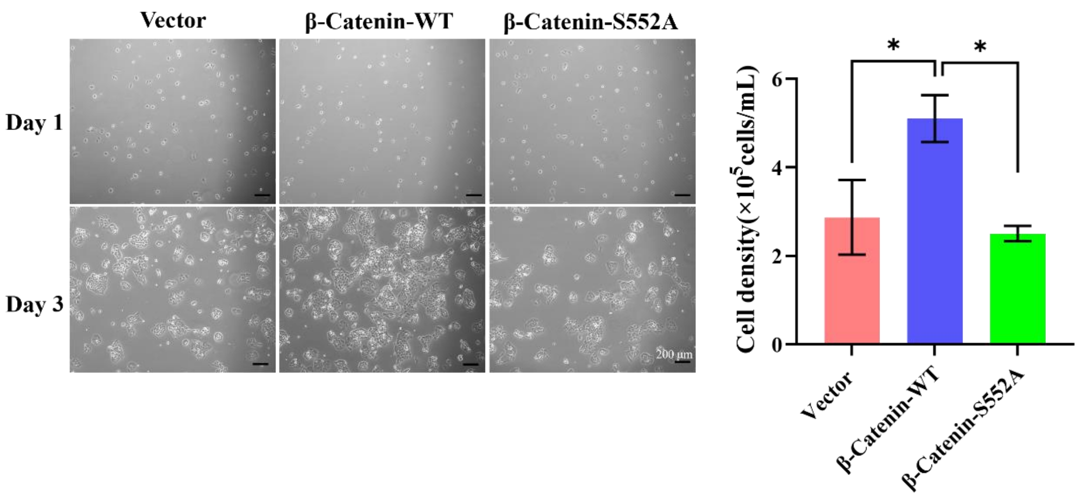

Figure S7. Cell proliferation was assessed through cell counting. U2OS cells (2×10^4 cells) were seeded in 6-cm cell culture dishes and cultured in GMF environments for 1 and 3 days, respectively. After 3 days of culture, the cells were harvested, resuspended in 1 mL of cell culture medium, and counted using an automatic cell

counter (Countstar BioTech) (n = 3). *p<0.05, **p<0.01, ***p<0.001.

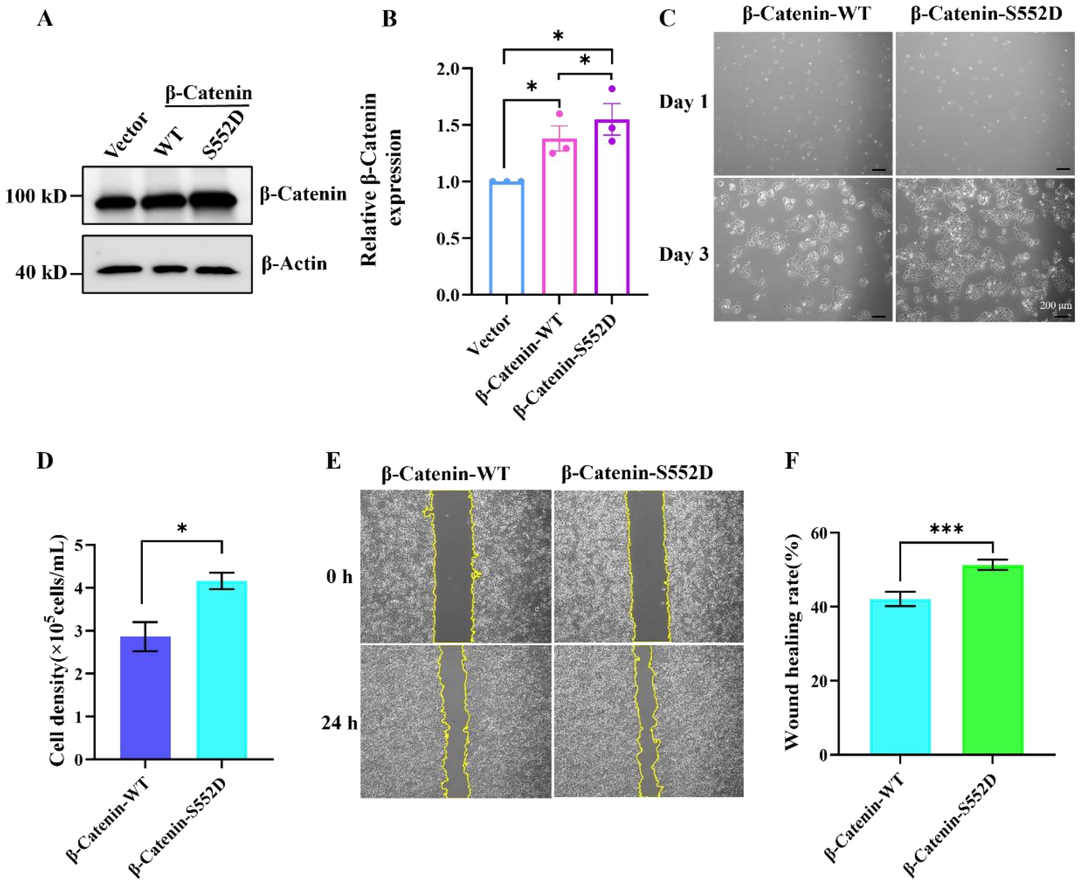

Figure S8. Overexpression of β-Catenin-S552D enhances the proliferation and migration of U2OS cells. (A) WB analysis of β-Catenin and β-Catenin-S552D expression levels in U2OS cells following lentiviral infection (n=3). (B) Quantification of relative β-Catenin expression levels in U2OS cells. (C) and (D) Cell proliferation assays conducted through cell counting (n=3). (E) and (F) Wound healing assays demonstrating the effects of overexpression of β-Catenin-WT and β-Catenin-S552D on U2OS cell migration (n=5). *p<0.05, **p<0.01, ***p<0.001.

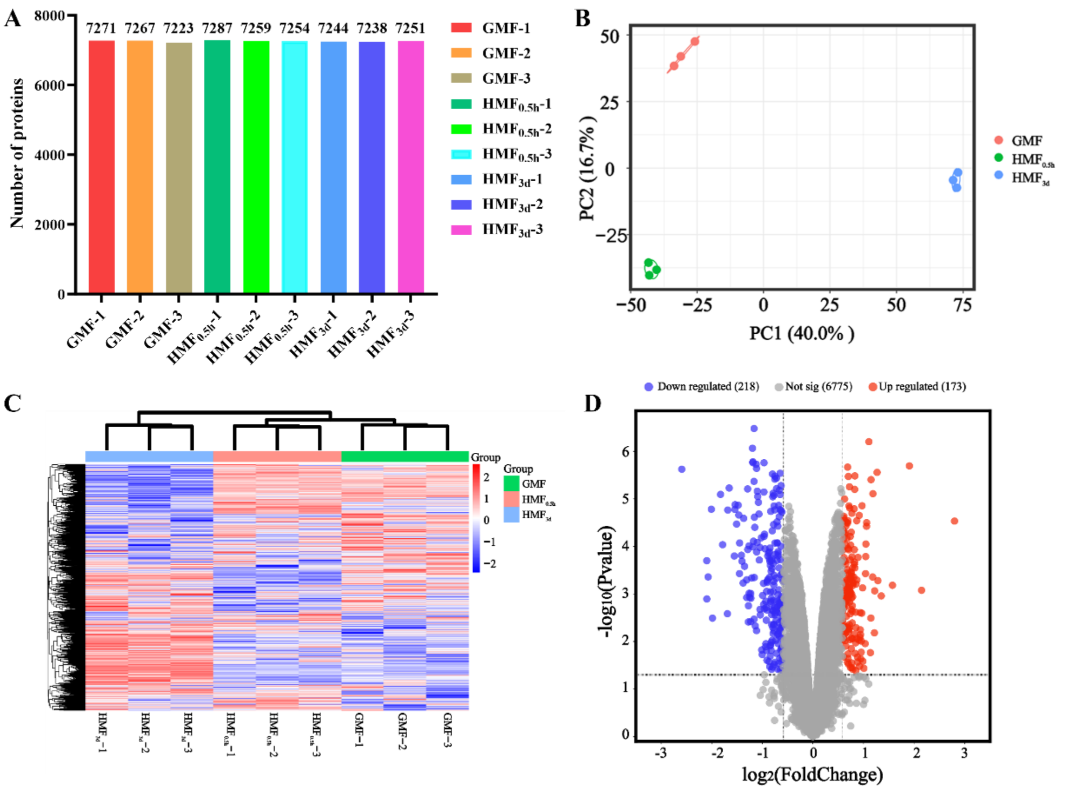

Figure S9. Proteomic analysis of U2OS cells cultured in GMF and U2OS cells exposed to a HMF for 0.5 hours and 3 days. (A) The number of proteins identified in each group. (B) Principal component analysis and hierarchical clustering analysis of the proteins identified in GMF, $HMF_{0.5h}$ and HMF_{3d} groups. (C) Heat map of z-scored protein abundances of the proteins differentially expressed through HMF exposure. (D) Volcano plots illustrating the numbers of upregulated and downregulated proteins between the GMF and HMF_{3d}.

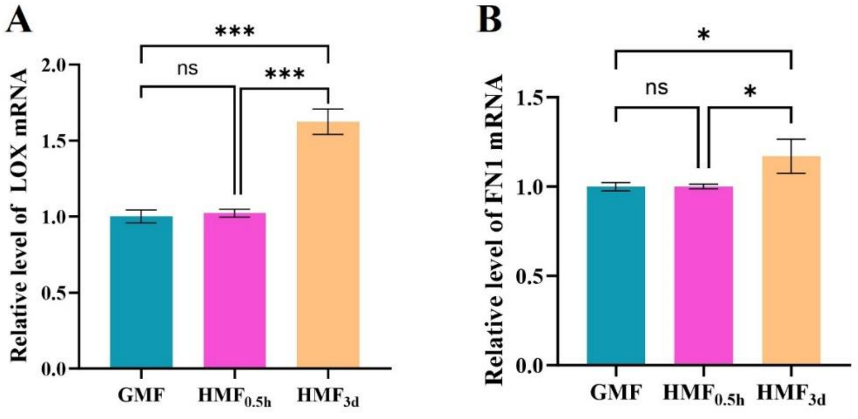

Figure S10. HMF exposure for 3 days enhances the transcription of *LOX* and *FN1*. (A) Relative mRNA levels of LOX in U2OS cells exposed to HMF for 0.5 hours and 3 days (n=3). (B) Relative mRNA levels of FN1 in U2OS cells exposed to HMF for 0.5 hours and 3 days (n=3). *p<0.05, **p<0.01, ***p<0.001.

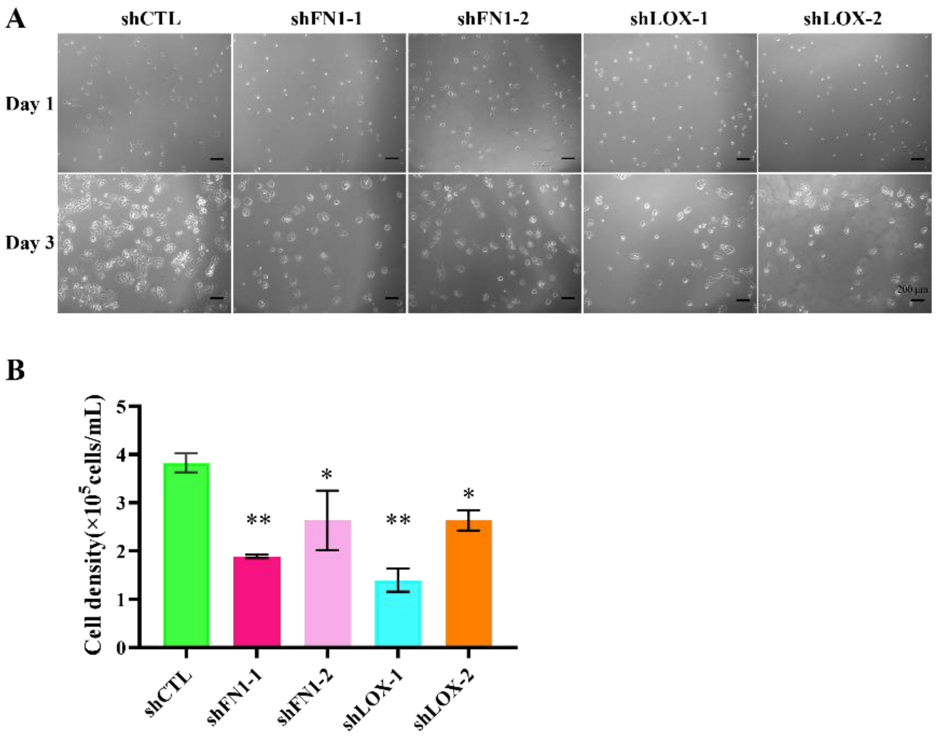

Figure S11. FN1 or LOX knockdown inhibits the proliferation of U2OS cells under GMF conditions. (A) U2OS cells (1.1×10^4 cells) were seeded in 6-cm cell culture dishes and cultured in GMF environments for 1 and 3 days, respectively. (B) After 3 days of culture, cells were harvested, resuspended in 1 mL of cell culture medium, and counted using an automatic cell counter (Countstar BioTech) (n=3). *p<0.05, **p<0.01, ***p<0.001.

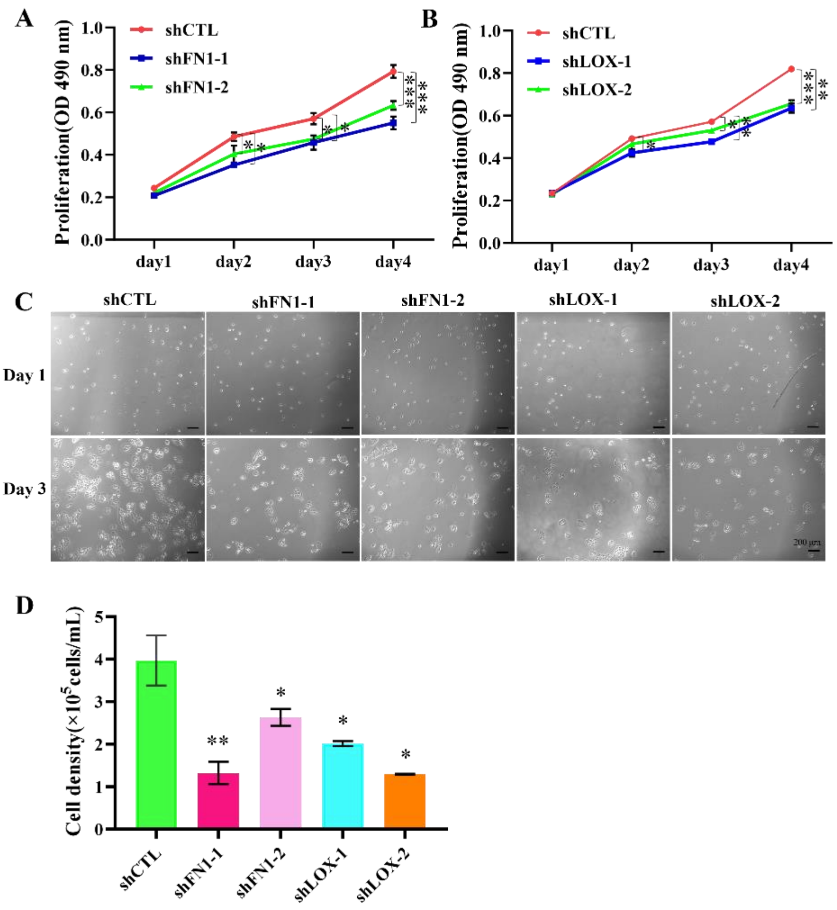

Figure S12. FN1 or LOX knockdown inhibits the proliferation of U2OS cells under HMF conditions. (A) and (B) MTS assay assessing the proliferation of cells treated with shRNA in HMF (n=4). (C) U2OS cells (1.1×10^4 cells) were seeded in 6-cm cell culture dishes and cultured in HMF environments for 1 and 3 days, respectively. (D) After 3 days of culture, cells were harvested, resuspended in 1 mL of cell culture medium, and counted using an automatic cell counter (Countstar BioTech) (n=3). *$p<0.05$, **$p<0.01$.

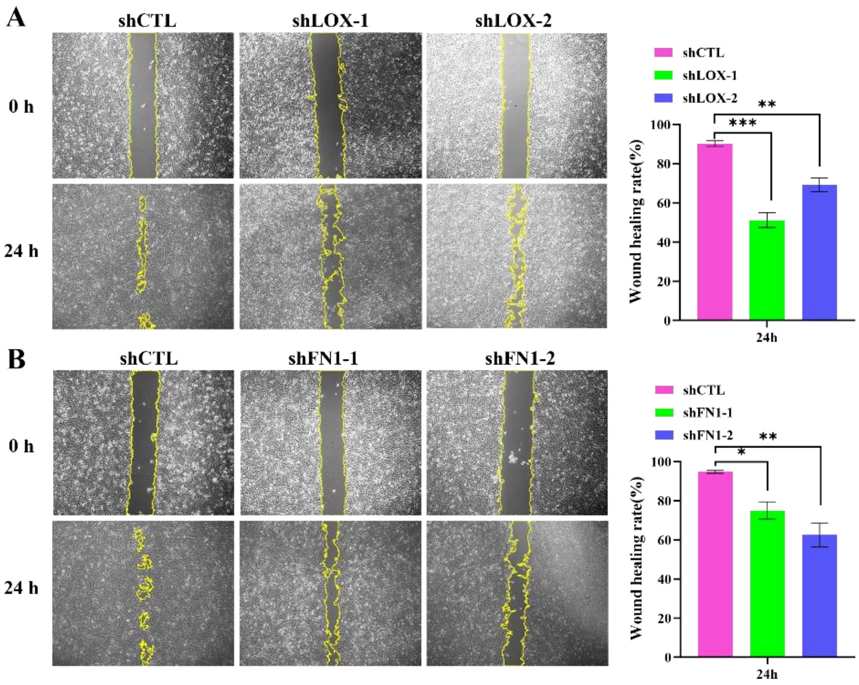

Figure S13. Knockdown of FN1 or LOX inhibits the migration of U2OS cells under HMF conditions. (A) Wound healing assay demonstrating the effects of LOX knockdown on U2OS cell migration in HMF (n=5). (B) Wound healing assay demonstrating the effects of FN1 knockdown on U2OS cell migration in HMF (n=5). *p<0.05, **p<0.01, ***p<0.001.

Table S1. The Sequences of shRNA Control and shRNA Targeting Human *FN1* and *LOX*

	**Sequence (5’-3’)**
ShRNA-Control (shCTL)	CCGGGAGGCTTCTTATAAGTGTTTACTCGAGTAAACACTTATAAGAA GCCTCTTTTTG
ShRNA-FN1#1 (shFN1-1)	CCGGGCTGAAGACACAAGGAAATAACTCGAGTTATTTCCTTGTGTCT TCAGCTTTTTG
ShRNA-FN1#2 (shFN1-2)	CCGGTGCAGCACAACTTCGAATTATCTCGAGATAATTCGAAGTTGTG CTGCATTTTTG
ShRNA-LOX#1 (shLOX-1)	CCGGCTCTGACGACAACCCTTATTACTCGAGTAATAAGGGTTGTCGT CAGAGTTTTTG
ShRNA-LOX#2	CCGGACTGCCAGTGGATTGATATTACTCGAGTAATATCAATCCACTG

(shLOX-2)	GCAGTTTTTG

Table S2. The relative level of serine phosphorylation at position 552 of the β-Catenin after both 0.5 hours and 3 days of exposure in HMF compared to GMF.

pS552-β-Catenin	FC	p-Value
$HMF_{0.5h}$ VS GMF	1.64	0.005
HMF_{3d} VS GMF	1.67	0.006

Table S3. Quantitative proteomic analysis of FN1 and LOX in U2OS cells cultured in HMF_{3d} compared to those cultured in GMF_{3d}.

Proteins	FC	p-Value
FN1	1.64	1.40116E-05
LOX	1.60	0.005239333

Note: FC, fold change

Table S4. The sequences of qPCR primers

Genes	Sequence (5'-3')
LOX	F: ACTGCACACACACAGGGATT

	R: AGCTGGGGTTTACACTGACC
FN1	F: GGTCCGGGACTCAATCCAAA
	R: GGTCCGGGACTCAATCCAAA
Actin	F: CTCTTCCAGCCTTCCTTCCT
	R: AGCACTGTGTTGGCGTACAG
